# Targeting mannose receptor expression on macrophages in atherosclerotic plaques of apolipoprotein E-knockout mice using ^111^In-tilmanocept

**DOI:** 10.1186/s13550-017-0287-y

**Published:** 2017-05-03

**Authors:** Zohreh Varasteh, Fabien Hyafil, Nadège Anizan, Devy Diallo, Rachida Aid-Launais, Sarajo Mohanta, Yuanfang Li, Miriam Braeuer, Katja Steiger, Jonathan Vigne, Zhengtao Qin, Stephan G. Nekolla, Jean-Etienne Fabre, Yvonne Döring, Dominique Le Guludec, Andreas Habenicht, David R. Vera, Markus Schwaiger

**Affiliations:** 1 Department of Nuclear Medicine, Klinikum rechts der Isar der TUM, Ismaningerstrasse 22, 81675 Munich, Germany; 2 Department of Nuclear Medicine, Hôpital Bichat, Paris, France; 3 Fédération de Recherche en Imagerie Multimodalité, Université Paris Diderot, Paris, France; 4 INSERM U1148 Laboratory of Vascular Translational Science, Paris, France; 5 Institute for Cardiovascular Prevention, University Hospital of Ludwig-Maximilians-University, Munich, Germany; 6 Institute of Pathology, Technische Universität München, Munich, Germany; 7 UCSD Moores Cancer Center, University of California, San Diego, La Jolla USA; 8 German Centre for Cardiovascular Research (DZHK), partner site Munich Heart Alliance, Munich, Germany

**Keywords:** Non-invasive imaging, Atherosclerosis, Inflammation, M2 differentiated macrophages, SPECT/CT, Tilmanocept

## Abstract

**Background:**

Atherosclerotic plaque phenotypes are classified based on the extent of macrophage infiltration into the lesions, and the presence of certain macrophage subsets might be a sign for plaque vulnerability. The mannose receptor (MR) is over-expressed in activated macrophages. Tilmanocept is a tracer that targets MR and is approved in Europe and the USA for the detection of sentinel lymph nodes. In this study, our aim was to evaluate the potential of ^111^In-labelled tilmanocept for the detection of MR-positive macrophages in atherosclerotic plaques of apolipoprotein E-knockout (ApoE-KO) mouse model.

**Methods:**

Tilmanocept was labelled with ^111^In. The labelling stability and biodistribution of the tracer was first evaluated in control mice (*n* = 10) 1 h post injection (p.i.). For in vivo imaging studies, ^111^In-tilmanocept was injected into ApoE-KO (*n* = 8) and control (*n* = 8) mice intravenously (i.v.). The mice were scanned 90 min p.i. using a dedicated animal SPECT/CT. For testing the specificity of ^111^In-tilmanocept uptake in plaques, a group of ApoE-KO mice was co-injected with excess amount of non-labelled tilmanocept. For ex vivo imaging studies, the whole aortas (*n* = 9 from ApoE-KO and *n* = 4 from control mice) were harvested free from adventitial tissue for Sudan IV staining and autoradiography. Cryosections were prepared for immunohistochemistry (IHC).

**Results:**

^111^In radiolabelling of tilmanocept provided a yield of greater than 99%. After i.v. injection, ^111^In-tilmanocept accumulated in vivo in MR-expressing organs (i.e. liver and spleen) and showed only low residual blood signal 1 h p.i. MR-binding specificity in receptor-positive organs was demonstrated by a 1.5- to 3-fold reduced uptake of ^111^In-tilmanocept after co-injection of a blocking dose of non-labelled tilmanocept. Focal signal was detected in atherosclerotic plaques of ApoE-KO mice, whereas no signal was detected in the aortas of control mice. ^111^In-tilmanocept uptake was detected in atherosclerotic plaques on autoradiography correlating well with Sudan IV-positive areas and associating with subendothelial accumulations of MR-positive macrophages as demonstrated by IHC.

**Conclusions:**

After i.v. injection, ^111^In-tilmanocept accumulated in MR-expressing organs and was associated with only low residual blood signal. In addition, ^111^In-tilmanocept uptake was detected in atherosclerotic plaques of mice containing MR-expressing macrophages suggesting that tilmanocept represents a promising tracer for the non-invasive detection of macrophages in atherosclerotic plaques.

## Background

Atherosclerosis and its complications remain the main cause of morbidity and mortality in industrialized countries [1]. Although the conventional risk factors are well understood and adequately treated, complications associated with atherosclerosis keep growing.

Macrophages, which are the main component of atherosclerotic plaques, are the first inflammatory cells to enter atherosclerotic lesions [2]. Studies of atherosclerosis reported that macrophages are involved at multiple stages of plaque formation [3]. Both classically (known as M1) and alternatively (M2) differentiated macrophages have been identified in atherosclerotic plaques. The phenotypic spectrum of macrophages in atherosclerotic plaques is, however, complex [3]. The in vivo detection of macrophage polarization in atherosclerotic plaques with imaging may pave the way for better understanding of the exact roles of M1 and M2 macrophages in different stages of the disease which is critical for the prognosis, diagnosis and treatment of atherosclerosis.

Several imaging techniques have been used for the more precise characterization of plaque composition [4]. Morphological features of atherosclerotic plaques can be detected using magnetic resonance imaging (MRI) in the carotids [5] or computed tomography angiography (CTA) in the coronary arteries [6]. However, molecular imaging offers the opportunity to look more in depth at the biology of atherosclerotic plaques and assess biological hallmarks involved in plaque vulnerability. ^18^FDG has become a mainstay of metabolic imaging in atherosclerosis but does not allow us to identify specifically the type and activation status of inflammatory cells taking up the radiotracer in the plaques [7–9]. Consequently, tracers that more specifically target biomarkers expressed on macrophage-rich cell infiltrates might facilitate the non-invasive detection of inflammatory activity in the vessel walls. However, the selection of a target biomarker in culprit atherosclerotic lesions remains challenging [10].

M2-oriented macrophages express carbohydrate-binding receptors, e.g. mannose receptor (MR), also known as CD206, is a highly effective endocytic C-type lectin receptor (175 kDa). Evidence for the presence of M2 macrophages in human atherosclerotic plaques was reported by Chinetti-Gbaguidi et al. [11]. Although some controversies exist [11–13], effective targeting of macrophages using MR-specific radioconjugates reported as a potential robust approach for imaging of high-risk atherosclerotic plaques [14].

**Fig. 1:**
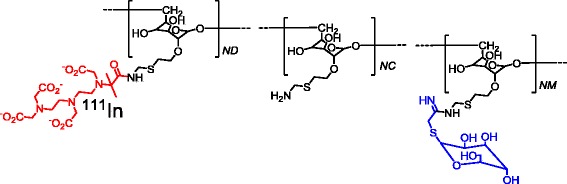
Chemical structure of tilmanocept. It is composed of a dextran backbone (*black*) and multiple units of d-mannose (*blue*) and DTPA (red) attached to the backbone. The d-mannose units are the receptor recognizing moieties, and the DTPA units are used for ^111^In labelling

Diethylenetriaminepentaacetic acid (DTPA)-mannosyl-dextran (tilmanocept) (Fig. 1) is a novel MR-targeting molecule with calculated average molecular weight ranging from 15 to 23 kDa [15] and molecular diameter of 7.1 nm [16]. Its ^99m^Tc-labelled analogue with trade name of Lymphoseek is the first radiopharmaceutical diagnostic imaging agent approved by the US FDA and the EU EMA for receptor-targeted lymphatic mapping to determine the pathologic nodal status in patients undergoing surgical treatment [16]. In vitro binding assays have shown subnanomolar binding affinity to MR expressed on dendritic cells (DC) [17] as well as on J774 E macrophages [18].

The aim of this study is to evaluate whether the presence of MR-positive (MR^+^) macrophages in atherosclerotic plaques can be detected in vivo with ^111^In-tilmanocept. After investigating the in vivo biodistribution of ^111^In-tilmanocept in control mice, in vivo and ex vivo imaging studies were performed in apolipoprotein E-knockout (ApoE-KO) mice for quantitative analysis.

## Methods

### Radiolabelling and in vitro stability test

Tilmanocept was labelled with ^111^In at room temperature. Briefly, 80 μl of ^111^InCl, 80 μl of 0.1 M ammonium acetate (NH_4_OAc) buffer (pH 5.5) and 10 μl (10 nmol) of tilmanocept solution (1 mM solution in water) were allowed to react for 30 min at room temperature. Radiochemical purity (RCP) of the tracer was determined by radio-instant thin layer chromatography (radio-ITLC). The 10 × 1 cm ITLC strips (Agilent Technologies) were spiked with a drop of radioactive sample and exposed to 0.1 M sodium citrate pH 5 mobile phase. In this system, ^111^In-tilmanocept remains at the origin, whereas free ^111^In migrates with the solvent front. (Rf (^111^In-tilmanocept) = 0–4 cm, Rf (^111^In) = 7–10 cm). Peaks were identified and compared by region of interest (ROI) analysis.

The in vitro labelling stability was evaluated using SDS-PAGE analysis (100 V constant) on NuPAGE 4-12% bis-Tris gels (Invitrogen) with the aims to check the release of ^111^In and transchelation of ^111^In to serum proteins. Briefly, 5 nmol of ^111^In-tilmanocept was incubated in 0.5 ml of murine serum for 90 min at 37 °C. After incubation, the samples were treated with NuPAGE® LDS sample buffer according to the manufacturer’s instructions and finally 0.05 nmol of sample loaded on the gel. ^111^In-acetate was loaded in a separate lane as a low molecular weight internal reference. After the analysis, the gels were dried, and radioactivity distribution along the gels was evaluated as described above.

### Animals

For biodistribution and in vivo specificity test, C57BL/6 mice (female, 7 week old, 19–22 g weight, from Charles River Laboratories) were used. Adult ApoE-KO mice (female and male, 40–48 weeks old, 25–32 g weight, from Jackson laboratory) which are known to develop extensive atherosclerotic lesions were used for in vivo and ex vivo imaging studies. Age-matched (female and male) C57BL/6 mice were used as controls. All mice were fed a normal chow diet. Experiments were approved by the local animal care committee and were in accordance with the German Animal Welfare Act.

### Biodistribution studies

Two groups (*n* = 5 per group) of female C57BL/6 mice were injected i.v. with 10 kBq of ^111^In-tilmanocept (total tilmanocept concentration was 1.5 pmol/g of body weight resulted in 28–33 g/mouse) to assess in vivo tracer stability, blood clearance and overall biodistribution of the compound. In order to test in vivo binding specificity, one group was co-injected with 10 nmol (more than 300-fold excess) of non-labelled tilmanocept. The mice were sacrificed 1 h p.i. by i.v. injection of high dose of pentobarbital (Narcoren). Blood was collected by heart puncture and organs were excised. The samples were put in the pre-weighed plastic vials. The samples were weighed, and their radioactivity was measured in an automated gamma counter (PerkinElmer 2480 WIZARD^2^) collecting 171 and 245 keV gamma rays (window width of 135–300 keV). The uptake in tissue and organs were calculated as percentage injected dose per gram of tissue (%ID/g). For the gastrointestinal tract and the carcass, injected activity per gram of whole sample was calculated (%ID/g).

### In vivo SPECT/CT imaging

Two groups of mice, ApoE-KO (*n* = 8) and controls (*n* = 8), were used for in vivo imaging studies. Mice were kept fully sedated with 1.5–2% isoflurane during injections and SPECT/CT imaging. SPECT/CT images were acquired using nanoSPECT/CT (Mediso medical imaging systems, Hungary) with four detectors after i.v. injection with 2–3 MBq of ^111^In-tilmanocept (0.4–0.6 nmol/mouse), 90 min p.i. Ultra-high resolution (0.6 mm) multi-pinhole whole-body mouse collimators were used. In addition, 20 angular projections with 40 s/projection and a multi-peak energy window (245 keV ± 20% and 171 keV ± 20%) were used. A group of ApoE-KO mice was co-injected with blocking dose (10 nmol) of non-labelled tilmanocept. Data were reconstructed using TeraTomo software (Mediso medical imaging systems, Hungary), and a 3D Monte Carlo-based algorithm was used (64 × 64 matrix, voxel size in 0.47 × 0.47 × 0.47 mm^3^, 3 subsets and 48 iterations).

### Ex vivo Sudan IV, autoradiography, histology and immunohistochemistry

For ex vivo imaging studies, two groups of mice, ApoE-KO (*n* = 9) and controls (*n* = 4), were injected with ^111^In-tilmanocept (2–3 MBq). A group of ApoE-KO mice (*n* = 5) was co-injected with blocking dose of non-labelled tilmanocept. After 90 min, the mice were euthanized with an overdose of isoflurane. The whole aortas (from the sinotubular junction to aortic bifurcation) were dissected out under the dissection microscope and rinsed with saline to remove the blood. All adventitial adipose tissue was removed by careful dissection. The aortas were opened longitudinally, mounted en face on a black wax surface to expose the luminal side and stained with Sudan IV for neutral lipids using a previously published method [19]. The images of en face Sudan IV-stained aortas were used to measure whole aorta surface areas. Briefly, the outer border of the whole aortas were encircled manually and their areas (mm^2^) were measured using a public domain software Image J (National Institutes of Health (NIH), USA) [19].

After Sudan IV staining, the opened aortas were exposed to phosphor imaging plates (FUJI IMAGING PLATE, FUJIFILM). After 7 days of exposure time, the imaging plates were scanned and the autoradiograms were obtained with a phosphor imaging system (raytest, Straubenhardt, Germany) and the images were analysed for count densities. The regions of interest (ROIs) were placed on the whole aorta, and total quantum level units (QL) that each area contains were measured. Data were used to calculate autoradiographic signal intensity (QL/mm^2^) in the whole aorta.

In addition, the excised aortas of ApoE-KO (*n* = 2) and control mice (*n* = 2) were frozen and cut into 8-μm-thick cryosections for immunohistochemistry (IHC). After pre-treatment with 4% formalin for 10 min, the sections were either stained with hematoxylin-eosin (HE) or immunostained. Macrophages were detected with rat anti-mouse CD107b (clone M3/84; BD Biosciences). MR was stained with a goat anti-mouse anti-MR/CD206 antibody (R&D systems; AF2535). Biotinylated secondary antibodies (rabbit anti-rat or rabbit anti-goat) were used for detection. Immunostains were visualized by streptavidin peroxidase and DAB (3,3′-diaminobenzidine) which gives a dark brown precipitate.

### Statistics

Data on biodistribution and autoradiography were analysed by unpaired, two-tailed *t* test using GraphPad Prism (version 4.00 for Windows GraphPad Software) in order to determine significant differences (*p* < 0.05).

## Results

### Radiolabelling and in vitro stability test

**Fig. 2:**
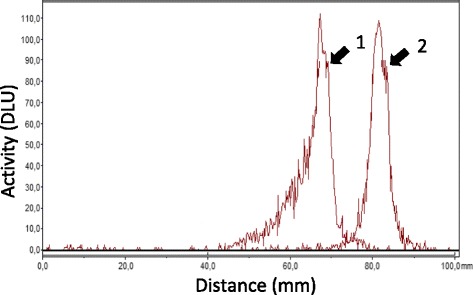
SDS-PAGE analysis of ^111^In-tilmanocept. *1* ^111^In-tilmanocept sample, which was incubated in murine serum at 37 °C for 90 min. *2* ^111^In-acetate, which was used as a low molecular weight radioactivity marker on the same gel

The labelling with ^111^In in the described conditions provided high yield. Tilmanocept was labelled with an overall RCP of 99.6 ± 0.2% with ^111^In and required no further purification for in vivo studies. The specific activity of 5 GBq · μmol^−1^ was obtained. SDS-PAGE analysis of the sample incubated in serum for 90 min at 37 °C showed a single radioactivity peak (Fig. 2). No peak corresponding to free or low molecular weight ^111^In was detected.

### Biodistribution studies

Data concerning the ex vivo biodistribution of ^111^In-tilmanocept in C57BL/6 mice are presented in Table 1. The biodistribution data showed rapid blood clearance of ^111^In-tilmanocept via excretory organs. High radioactivity accumulation was observed in the liver, pancreas, spleen and kidneys. Specific uptake was observed in the heart, salivary glands and most of the abdominal area organs, i.e. liver, spleen and pancreas. The pre-saturation of MR by co-injection of non-labelled tilmanocept decreased the uptake of ^111^In-tilmanocept 1.5- to 3-fold in the above-mentioned organs. Radioactivity accumulation in the kidneys increased in the blocked group. This is consistent with a high density of MR residing in renal mesangial cells [20].

**Table 1:** Biodistribution of ^111^In-tilmanocept

Organs	Non-blocked	Blocked
Blood	0.18 ± 0.02	0.2 ± 0.1
Heart	2.2 ± 0.3	1.22 ± 0.05*
Stomach	3.4 ± 0.4	1.7 ± 0.3*
Lung	1.2 ± 0.2	1.1 ± 0.1
Salivary glands	4.4 ± 0.5	1.8 ± 0.2*
Liver	22.6 ± 2.7	14.7 ± 1.3*
Pancreas	3.4 ± 0.2	1.2 ± 0.1*
Spleen	10.4 ± 2.0	6.2 ± 0.3*
Small intestine	1.9 ± 0.8	1.6 ± 0.5
Large intestine	1.3 ± 0.2*	2.5 ± 0.2
Fat	1.02 ± 0.58	0.24 ± 0.03
Skin	1.3 ± 0.2	1.2 ± 0.3
Muscle	0.7 ± 0.2	0.50 ± 0.04
Bone	2.5 ± 0.5	2.2 ± 0.7
Kidney	23.2 ± 5.7*	55.7 ± 4.0
Carcass	14.2 ± 1.7*	22.2 ± 1.1
GI	3.8 ± 1.7	2.9 ± 0.2
Aorta^a^	0.005 ± 0.000	N/A

Biodistribution of ^111^In-tilmanocept (1.5 pmol/g of body weight) in female C57BL/6 mice, 1 h p.i. Blocked group was co-injected with blocking dose (10 nmol) of non-labelled tilmanocept. Data are presented as the mean percentage of injected dose per gram of tissue (%ID/g ± SD) except in carcass, gastrointestinal tract (GI) and aortas (dissected only from non-blocked mice, *n* = 2) where the uptakes are presented as %ID per whole sample.

*N/A* not applicable

*Show significantly lower uptake (*p* < 0.05)

^a^Whole aortas from the sinotubular junction to aortic bifurcation were collected

### In vivo SPECT/CT imaging

**Fig. 3:**
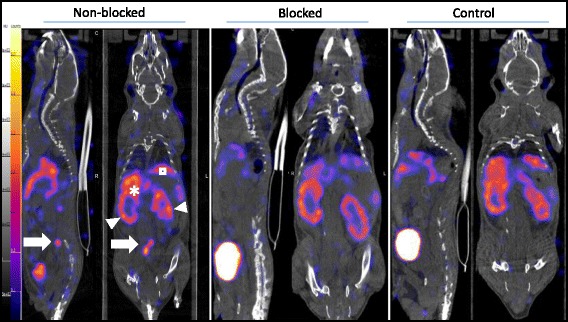
Sagittal and coronal views of SPECT/CT images acquired in vivo 90 min after i.v. injection of ^111^In-tilmanocept from ApoE-KO non-blocked, blocked and control mice. Note the intense focal signals in low abdominal atherosclerotic plaques of ApoE-KO non-blocked mice (*white arrows*). In contrast, no focal uptake was detected in the aortas of ApoE-KO mice after blocking with excess amount (10 nmol) of non-labelled tilmanocept and in those of control mice. Kidneys (*arrowheads*) liver (*asterisk*), spleen (*square*)

Images acquired 90 min p.i. with ^111^In-tilmanocept for ApoE-KO non-blocked and blocked as well as control mice are presented in Fig. 3. As expected, the highest accumulation of the radioactivity was observed in the abdominal area receptor-positive organs [21], e.g. liver, spleen and kidneys. The lesions in the lower part of the abdominal aorta before it branches into the paired iliac arteries were clearly visualized in ApoE-KO non-blocked mice. The uptake of ^111^In-tilmanocept decreased significantly in the liver of the ApoE-KO mouse co-injected with excess amount of non-labelled tilmanocept.

### Ex vivo Sudan IV, autoradiography, histology and immunohistochemistry

**Fig. 4:**
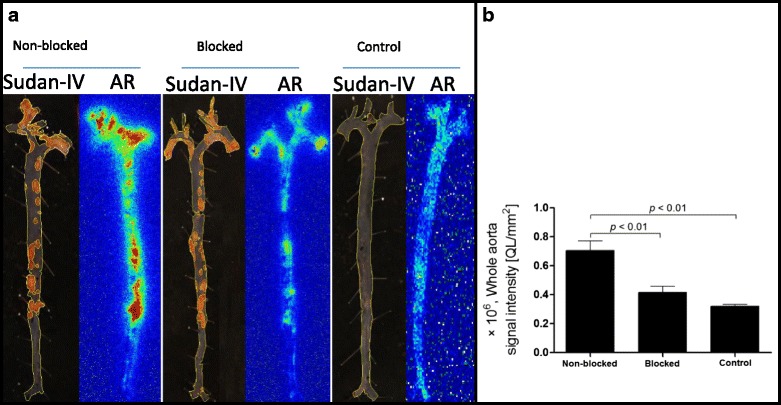
**a** Sudan IV staining and corresponding autoradiography (AR) of the aortas extracted from ApoE-KO non-blocked, blocked and control mice. Whole aorta surface area was quantified using ImageJ software. **b** Quantification of the autoradiography signals expressed as intensity per unit area of the whole aorta (whole aorta autoradiographic signal/whole aorta area, QL/mm^2^)

The results of Sudan IV staining and autoradiography of aortic samples dissected out from ApoE-KO non-blocked, blocked and control mice showed a clear superimposition of radioactivity deposition and atherosclerotic lesions (Fig. 4a). Morphometric data from representative image of the Sudan IV staining showed more plaques in the aortic arc and low abdominal aorta which corroborates the intensive signal from autoradiography. The aortas from control mice did not exhibit focal lipid staining and radioactivity signal.

The results of quantification of the autoradiography images comparing signal intensity in the whole aortas are presented in Fig. 4b. The overall uptake intensity of ^111^In-tilmanocept was significantly (*p* < 0.01) greater in the aortas isolated from ApoE-KO non-blocked mice ((0.71 ± 0.07) × 10^6^, QL/mm^2^) compared to the ones isolated from WT mice ((0.32 ± 0.01) × 10^6^, QL/mm^2^). This uptake was receptor-mediated. Pre-saturating the receptors with co-injection of non-labelled tilmanocept decreased the uptake intensity almost 2-fold ((0.41 ± 0.04) × 10^6^, QL/mm^2^).

**Fig. 5:**
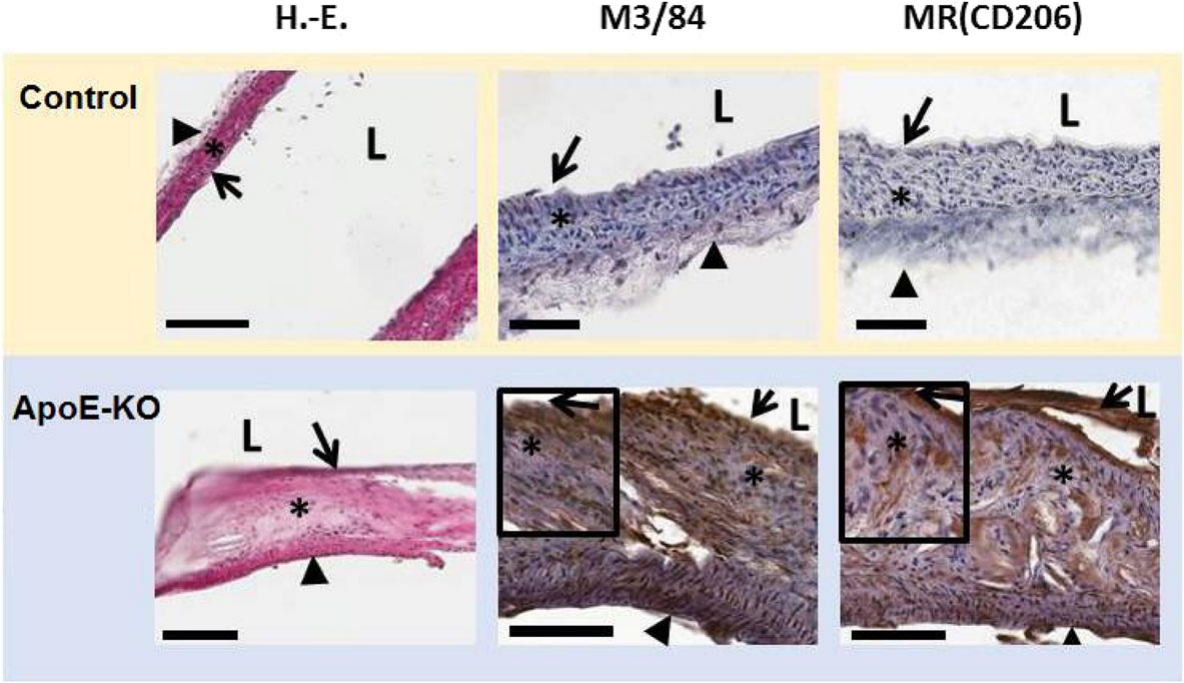
Representative images of HE, M3/84 (macrophages) and MR (CD206) immunohistochemistry. In control mice, no plaques with macrophages were observed, while fibrous/fibroatheromatous plaques were present in the aortas extracted from ApoE-KO mice. The lesions (fatty streaks and fibrous plaques) showed high amounts of MR^+^ macrophages (100 μm (*bars*), vascular lumen (*L*), intima (*arrow*), media (*asterisk*), adventitia (*arrowhead*))

Histological and immunohistochemical investigations revealed the presence of fibrous/fibroatheromatous plaques in the aortas isolated from ApoE-KO mice. Macrophages located in the fibrous cap layer of the lesions showed high amounts of MR expression confirmed by IHC (Fig. 5).

## Discussion

In this study, we demonstrated that the expression of MR in atherosclerotic plaques can be detected in vivo with SPECT imaging after i.v. injection of ^111^In-tilmanocept. Despite the small dimension of atherosclerotic plaques, lesions in the low abdominal aorta were successfully visualized with a dedicated animal SPECT/CT, 90 min p.i. The high and specific uptake of the tracer in atherosclerotic plaques was also confirmed ex vivo by autoradiography and associated with the presence of high number of MR^+^ macrophages in the lesions.

Studies of macrophage polarization in vitro and in mostly mouse models of atherosclerosis have led to a simplified view that M1 macrophages are facilitating the formation of the necrotic core and plaque destabilization, whereas M2 macrophages have anti-atherogenic properties mediating plaque stability [22]. However, the phenotypic spectrum of macrophages in vivo is more complex. For instance, intermediate spectra of macrophage differentiation states were reported by Finn et al. [23]. In addition, the exact role of M2 macrophages in atherosclerosis and their contribution to vulnerable plaque formation is still controversial. M2 polarization of macrophages induced after incubation with IL-4 was associated with over-expression of receptors involved in phagocytosis translating into enhanced phagocytic activities. In addition, M2 macrophages present low capacity to ingest native and oxidized lipoproteins, suggesting that they are less susceptible to become foam cells [11]. The ability of M2 macrophages to clear apoptotic cells without accumulating lipids suggests that they may have beneficial roles in atherosclerotic lesion development [11]. On the other hand, Mauriello et al. reported that the features of asymptomatic thrombotic plaques in the human carotid artery are not completely consistent with the morphological definition of a vulnerable plaque and M2 but not M1 macrophages were located in the relatively thicker fibrous cap near the rupture site, suggesting that M2 macrophages might also modulate the process of plaque rupture [24]. In addition, Tahara et al. reported high density of M2 and MR^+^ macrophages in the high-risk plaques obtained from subjects who had experienced sudden cardiac death [14]. Although further investigations regarding the roles of M1 and M2 macrophages in atherosclerotic lesions are needed, it is clear that visualization of the exact macrophage phenotypes present in the plaques is critical for the prognosis, diagnosis and treatment of atherosclerosis.

Different targeted nuclear imaging probes have been developed against MR. The radiolabelled single-domain antigen-binding fragments derived from Camelidae heavy-chain antibodies (known as nanobodies) specifically targeting the macrophage MR have shown high potential for clinical implementation for imaging of the tumour-promoting macrophages [25, 26] and rheumatoid arthritis [27]. Effective targeting of imaging agents to MR-expressing cells is a potential approach for atherosclerotic plaque imaging. The feasibility of imaging of plaques with ^18^F-labelled d-mannose (2-deoxy-2-[^18^F]fluoro-d-mannose, ^18^F-FDM) was reported by Tahara et al. [14]. They have demonstrated that ^18^F-FDM uptake is non-inferior to ^18^F-FDG uptake for imaging of plaque inflammation [14]. Kim et al. reported the feasibility of ^68^Ga-labelled NOTA-coupled mannosylated human serum albumin to non-invasively detect M2 macrophages in vulnerable atherosclerotic plaques [28]. In this study, our aim was to assess the potential of tilmanocept for in vivo imaging of the macrophages in atherosclerotic plaques of ApoE-KO mice.

Tilmanocept, which is of similar size to the nanobodies (12–15 kDa), was originally designed for targeted lymphatic mapping and guiding sentinel lymph node (SLN) biopsy in patients with solid tumours and has shown subnanomolar affinity to MR [17, 18]. It carries multiple units of d-mannose (12–20 units [15]) for receptor recognition and DTPA chelators (3–8 units [15]) for radiolabelling (Fig. 1). The molecule has been labelled with ^68^Ga [29] and ^99m^Tc [30–32]. It was administrated peritumoural [30, 31], intradermal [31, 32], subareolar [32] and intraprostatic [29]. The intravenous administrations of ^68^Ga- and ^99m^Tc-tilmanocept were also reported [17, 33]. However, detailed biodistribution and in vivo stability data were not disclosed. Based on the reported data, ^99m^Tc-tilmanocept showed relatively slow blood clearance. The radioactivity concentration in the blood dropped from 6.7 ± 6.4%ID/g (1 h p.i.) to 1.33 ± 0.17%ID/g (2 h p.i.) for ^99m^Tc-tilmanocept [17] while 0.18 ± 0.02%ID/g blood radioactivity concentration was measured in this study for ^111^In-tilmanocept already 1 h p.i. This may be explained by relatively stable complexation of ^111^In with DTPA. The transchelation from radionuclide-DTPA complex to plasma proteins (e.g. transferrin) will result in longer blood pool circulation, and the excess radioactivity remaining in the blood will contribute to the background signal. It is known that ^111^In forms highly stable octadentate complex with DTPA. However, in the tilmanocept molecule, one of the carboxylic acid groups has been used to link DTPA molecule to the dextran backbone (Fig. 1) and four are retained for binding ^111^In. Despite relatively less stability of heptadentate ^111^In-DTPA complex compared to an octadentate complex, it has been used for ^111^In labelling of intact monoclonal antibodies and their fragments as well as peptides [34]. ^68^Ga- and ^99m^Tc-DTPA are reported to be less stable in serum [35, 36]. Therefore, in this study, we chose ^111^In for radiolabelling of tilmanocept for in vivo and ex vivo imaging studies.

This study shows some limitations. Regardless of its sufficient labelling stability with DTPA, ^111^In is associated with photon energies that are suboptimal for in vivo imaging and long half-life resulting in relatively high radiation burden, which both represent significant limitations for the translation of this radiolabelling approach to clinical studies. In addition for in vivo imaging studies, higher radioactivity doses needed to be injected in order to have a sufficient signal with multi-pinhole SPECT, which has an intrinsic low sensitivity, and this translated into the need of higher doses of tilmanocept. High hepatic signal after injection of ^111^In-tilmanocept might have hampered the in vivo detection of radiotracer uptake in the thoracic aorta because of the small size of the mouse body. The lower abdominal aorta plaques were visualized with higher contrast. Finally, ^111^In-tilmanocept uptake could be detected in this study in aortic atherosclerotic plaques of a mouse model. Whether radiolabelled tilmanocept accumulates also in complex human atherosclerotic plaques will need to be validated in future studies.

## Conclusions

In this study, we confirm in mice that tilmanocept radiolabelled with indium-111 accumulates in macrophage-rich organs after i.v. injection. In addition, ^111^In-tilmanocept uptake was detected in vivo and ex vivo in atherosclerotic plaques containing MR^+^ macrophages. Hence, radiolabelled tilmanocept represent a promising radiotracer for the detection of MR-expressing macrophages in atherosclerotic plaques. Future studies will aim at developing a stable PET radiolabelling of tilmanocept for the clinical translation of this radiotracer in order to associate high image quality and low radiation exposure.
